# Implementing and Evaluating Cognitive Stimulation Therapy for People Living with MCI or ADRD in a Community‐Based Organization

**DOI:** 10.1002/alz70858_101029

**Published:** 2025-12-25

**Authors:** Stephani Shivers, Olivia Cohen, Vicki Zimmer, Amy Puca, Edward Cisek

**Affiliations:** ^1^ CaringKind, New York, NY, USA; ^2^ Via Evaluation, Buffalo, NY, USA

## Abstract

Community‐based organizations (CBOs) meet a plethora of older adult needs in contemporary society including programs for individuals living with Mild Cognitive Impairment, Alzheimer's and other dementias. With an established presence, trust and growing number of caregiving, health promotion and chronic disease management programs in place, CBOs are well positioned to implement evidence‐based interventions (EBI) in the real world. With funding by the Administration for Community Living, Alzheimer's Disease Program Initiative, CaringKind, a dementia focused CBO, implemented evidence‐based Cognitive Stimulation Therapy (CST) and evaluated program and participant outcomes. CST is a globally practiced small group multi‐session EBI with demonstrated impact on cognition, depression and quality of life. Fourteen themed sessions delivered virtually or in‐person by trained facilitators actively stimulate cognitive processes through dialogue and multi‐sensory based activities. CaringKind's pre/post preliminary program data shows 76% (*n* = 153) of participants were ‘completers’ (attending 12+ program sessions); 48% of participants (*n* = 97) had decreased signs of depression on the Geriatric Depression Scale and 20% (*n* = 19) moved out of the depressed range; and 56% (*n* = 57) had increased hope and resilience on the Positive Psychology Outcomes Measure. Participant (*n* = 37) and proxy report (*n* = 38) qualitative surveys reveal positive impact on meeting current needs, connecting to peers, and perceived benefit for participant's future. Final data and outcomes will be presented at AAIC 2025. Lessons learned about barriers/facilitators to identify and enroll individuals in real‐world dementia programs will be shared, as well as feasibility challenges associated with finding, training and funding qualified staff to deliver sustainable evidence‐based programs as a CBO and service provider.

